# Retraction: Oncogenic Fibulin-5 Promotes Nasopharyngeal Carcinoma Cell Metastasis through the FLJ10540/AKT Pathway and Correlates with Poor Prognosis

**DOI:** 10.1371/journal.pone.0227931

**Published:** 2020-01-09

**Authors:** The PLOS ONE Editors

After publication of this article [1], it was reported that the Hone1 cell line, used in the experiments shown in Figs 3–9, is contaminated with HeLa cells [2]. In light of this issue the results reported for this cell line may not be representative of nasopharyngeal carcinoma cell biology. In the *PLOS ONE* article, the authors provided results from experiments on TW01 cells in Supporting Information files S1-S3 to support the Hone1 results shown in Figs 4, 6, and 8A, respectively [1]. TW01 data for other experiments reported in the article are available from the authors.

The authors noted that in preparing the figures for this publication [1], the wrong images were presented for the U2-OS cell panel of Fig 3B and shFibulin-5-Hone1/invasion panel of Fig 6D.

In Fig 3B, the images shown as representing fibulin-5 expression in U2-OS cells were instead obtained when examining fibulin-5 expression in a different head and neck cancer cell line.

In Fig 6D, the image shown as representing invasion results for shFibulin-5-Hone1 cells reports data from a different experiment (invasion in siRap-1A-FaDu cells) reported in [3], published 2013 [copyright American Society for Investigative Pathology; published by Elsevier Inc.], and which are not offered under a CC-BY license. This image is therefore excluded from this article's [1] license. At the time of retraction, the article [1] was republished to note this exclusion in the Fig 6 legend and the article’s copyright statement. Replication data for this experiment and the raw data underlying graphs in Fig 6D are available from the authors; the authors noted that quantification data shown in the original version of Fig 6D were obtained using the correct data.

There are several concerns about the western blot figures reported in [1]:

- In some cases (e.g. in Fig 9), it is difficult to evaluate the data due to the poor image quality.
- For all western blot panels in the article, the background is unexpectedly uniform.
- In Fig 1C, when adjusted for brightness and contrast, there appear to be vertical discontinuities between lane 3 and 4 of the Fibulin-5 blot, and there appear to be horizontal discontinuities above and below the bands in all lanes of the Fibulin-5 blot, suggesting that a blot image fragment was superimposed on a gray background image.
- In Fig 3C, there are visible splice lines around the bands for the Fibulin-5 Cytoplasm panel.
- In Fig 4A, when levels are adjusted there appears to be a vertical discontinuity before lane 2 and we cannot verify whether image data is present in lane 1 (Vehicle control).
- In Fig 6A, when adjusted for brightness and contrast, there appear to be vertical and horizontal discontinuities around the band in lane 1 of the Fibulin-5 blot, and we cannot verify whether image data is present in lanes 2, 3. There also appear to be horizontal discontinuities above and below the β-actin bands.
- In Fig 7B, when adjusted for brightness and contrast there appear to be vertical discontinuities in the image between lanes 3 and 4 in all three Hone1 blots, and there also appear to be horizontal discontinuities above and below the bands at different positions in lanes 1–3 versus lane 4 for each panel.
- In Fig 8B, when levels are adjusted, there are discontinuities in background above and below the bands, and the background for lane 1 appears different than the background for lanes 2, 3. There also appear to be discontinuities above and below the β-actin bands.

The authors noted that the original image data to support the western blot results are no longer available, and that the original data were cropped and/or rearranged, and were superimposed on a gray background image for the purpose of presentation. In light of the data unavailability, we are unable to resolve the above concerns and as such, the reliability and validity of the results are in question.

The authors provided data for a replication of the Fig 3C experiment, but the data provided for this experiment did not appear to be complete uncropped and unadjusted blot images, did not include a supernatant fraction, and the results of nuclear vs. cytoplasm blots did not demonstrate an enrichment in the nucleus as was reported in the Results of [1].

In light of the unresolved concerns discussed above, the *PLOS ONE* Editors retract this article.

CC and FF agreed with the retraction. LYS, LJS, TC, YY, WW, SY, CCH, CFH, YZ, HT, TH did not reply or could not be reached.
